# Coronary Microvascular Function and Beyond: The Crosstalk between Hormones, Cytokines, and Neurotransmitters

**DOI:** 10.1155/2015/312848

**Published:** 2015-06-01

**Authors:** Carlo Dal Lin, Francesco Tona, Elena Osto

**Affiliations:** ^1^Department of Cardiac, Thoracic and Vascular Sciences, University of Padua, Via Giustiniani 2, 35100 Padua, Italy; ^2^Centre for Molecular Cardiology, University of Zurich and University Heart Center, Department of Cardiology, University Hospital, Raemistrasse 100, 8091 Zurich, Switzerland

## Abstract

Beyond its hemodynamic function, the heart also acts as a neuroendocrine and immunoregulatory organ. A dynamic communication between the heart and other organs takes place constantly to maintain cardiovascular homeostasis. The current understanding highlights the importance of the endocrine, immune, and nervous factors to fine-tune the crosstalk of the cardiovascular system with the entire body. Once disrupted, this complex interorgan communication may promote the onset and the progression of cardiovascular diseases. Thus, expanding our knowledge on how these factors influence the cardiovascular system can lead to novel therapeutic strategies to improve patient care. In the present paper, we review novel concepts on the role of endocrine, immune, and nervous factors in the modulation of microvascular coronary function.

## 1. Coronary Microcirculation and Coronary Flow Reserve: Definition and Clinical Relevance

Obstructive disease of the epicardial coronary arteries was recognized as the cause of angina pectoris more than 2 centuries ago, and sudden thrombotic occlusion of an epicardial coronary artery has been established as the cause of acute myocardial infarction since over 100 years [1]. Cardiovascular diseases such as stable and unstable angina, acute myocardial infarction, peripheral artery disease, and stroke may be related to the loss of the protective properties of the endothelium, which in normal conditions preserves vascular tone, and inhibit thrombosis and inflammation [2]. Endothelial dysfunction, an early and reversible event in the pathogenesis of atherosclerosis, is associated with increased vascular smooth muscle tone, arterial stiffening, and intima-media thickness. As reported by Hirata et al., recent studies have shown that the severity of endothelial dysfunction correlates to the risk of primary or recurrent cardiovascular events. Moreover, a growing number of interventions known to reduce cardiovascular risk, such as physical exercise or alimentation, also improve endothelial function [3, 4]. Thus, the authors conclude that it is possible to consider the endothelial function as a “barometer” of cardiovascular health useful to direct patient management and evaluation of therapeutic strategies. The coronary microvasculature (vessels <300 *μ*m in diameter) cannot be directly imaged* in vivo*, but a number of invasive and noninvasive techniques can be used to assess parameters that depend directly on coronary microvascular function [5]. Among other vascular beds, the endothelial function can be assessed also at the level of the coronary circulation by mean of the coronary flow reserve (CFR) [6]. From a pathophysiological point of view, reduced CFR can result from the combination of different alterations such as impaired vasodilation, enhanced vasoconstrictor responsiveness, and/or structural remodeling of the coronary microvasculature. Thus, the functional status of the coronary microcirculation can be assessed by testing endothelium-dependent and endothelium-independent vascular responses [7]. Adenosine, dipyridamole, and papaverine are often used to trigger arteriolar vasodilation and hence increase coronary blood flow, mainly by a direct relaxing effect on vascular smooth muscle cells (endothelium-independent effect) [8]. On the other hand, acetylcholine (Ach) administration causes either vasodilation in the presence of a healthy endothelium able to stimulate nitric oxide (NO) production or vasoconstriction via the stimulation of muscarinic receptors on vascular smooth muscle cells [9] in the absence of a functional endothelium. Like Ach, also bradykinin and substance-P are mediators commonly used to test endothelial-dependent vasorelaxation [10].

**Figure 1:**
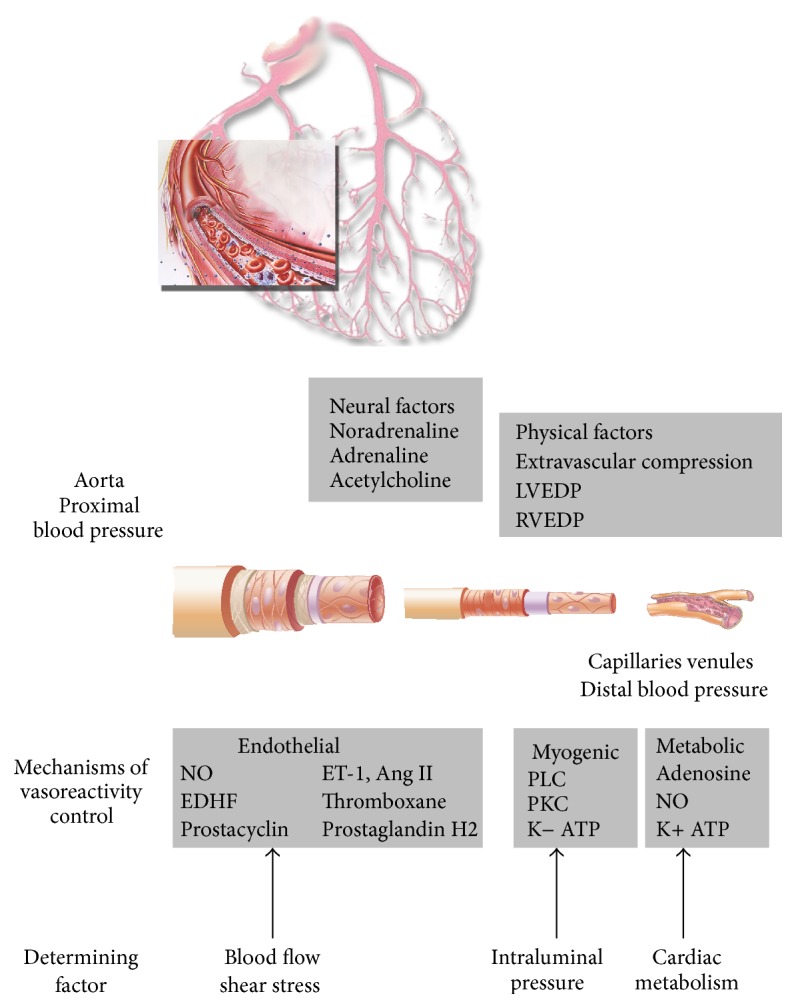
Physical, metabolic, and neural factors modulate microvascular coronary blood flow [9]. The coronary blood flow (CBF) is driven by the pressure difference between the aortic sinus and the coronary sinus (or the right atrium pressure). In the absence of obstructive stenoses, the epicardial arteries offer very little (10%) resistance to CBF and serve mainly as conductance vessels. Capillaries and venules are likewise responsible for only 10% of CBF resistance and mainly function as capacitance vessels, holding 90% of the total myocardial blood volume. Under normal conditions and to a large extent also under pathological conditions, coronary vascular resistance is primarily controlled by the prearterioles (vessels of 500 mm in diameter) and arterioles (200 mm). The prearterioles are epicardial (extramyocardial) vessels that react to changes in shear stress and intravascular pressure to preserve adequate perfusion pressure in the distal arteriolar bed. They are responsible for 25% of the total coronary vascular resistance. The arterioles are the true intramyocardial regulatory component of the coronary circulation and these vessels represent the largest proportion (55%) of the total coronary vascular resistance. Endothelium-dependent vasoreactivity prevails in the larger arterioles (100–200 mm in diameter) and translates flow-related stimuli into vasomotor responses, that is, vasodilation with increase in flow and vice versa. Medium-sized microvessels (40–100 mm in diameter) react predominantly to intraluminal pressure changes sensed by stretch receptors located in vascular smooth muscle cells (myogenic control, through signals mediated by phospholipase C and protein kinase C and calcium homeostasis); that is, they constrict when the intraluminal pressure increases and, conversely, dilate when the pressure decreases. Finally, the tone of the smaller arterioles (vessels of 40 mm in diameter) is modulated by the metabolic activity of the myocardium. As such, increased metabolic activity leads to vasodilatation of the smaller arterioles, which leads to pressure reduction in the medium-sized microvessels and myogenic dilation, which, in turn, increases flow upstream resulting in endothelium-dependent vasodilation. These mechanisms effectively and efficiently allow the microcirculation to regulate myocardial perfusion both at rest and at different levels of myocardial metabolic demand. LVEDP: left ventricular end diastolic pressure; RVED: right ventricular end diastolic pressure; ET-1: endothelin-1; ANG II: angiotensin II; NO: nitric oxide; EDHF: endothelial derived factor; PLC: phospholipase C; PKC: protein kinase C (modified from [9]).

It is important to notice that a high hemoglobin value, a major determinant of whole blood viscosity, predicts cardiovascular events [11]. Erythrocyte deformability is a key rheologic feature to allow blood flow, especially in the capillaries. Sandhagen and Lind [12] evaluated the relationships between blood viscosity, erythrocyte deformability, coronary risk, and endothelial vasodilatory function: rheological factors (such as heightened plasma viscosity and increased red blood cell aggregation) modify blood fluidity. A reduced fluidity may limit the microcirculatory flow due to the viscus resistance [13].  Figure 1 summarizes the key factors which contribute to the coronary microvascular function.

Coronary anatomy and myocardial blood flow are major determinants of clinical symptoms and survival in patients with epicardial coronary artery and coronary microvascular diseases (as in case of X syndrome or “stress cardiomyopathy”) [14–17]. The CFR, defined as maximal hyperemic flow divided by resting flow, is measured by echocardiography and by other techniques (coronary angiograms and fractional flow reserve, positron emission tomography, and magnetic resonance imaging), each one with distinct advantages and limitations [2]. An important distinction needs to be made between techniques that directly measure coronary blood flow (e.g., positron emission tomography) and those measuring blood flow velocity (e.g., Doppler catheters), from which coronary velocity reserve is only calculated [18].

CFR represents the ability of the coronary flow to increase above its basal value when the coronary vascular bed is maximally dilated. It is a global parameter of coronary flow, which is early altered in the presence of a coronary microvascular dysfunction/disease or epicardial coronary artery stenosis. It is possible to study coronary flow in all main coronaries by transthoracic Doppler echocardiography; however, normally the left anterior descending artery is the coronary of choice. CFR is defined as the ratio of maximal hyperemic to basal diastolic coronary velocity. Maximal hyperemic flow is obtained during adenosine infusion. Under normal physiological conditions, *α*-adrenergic vasoconstriction in the heart is suppressed by myogenic, endothelial, or metabolic factors, with the most important being NO [19]. Buus et al. demonstrated in healthy subjects that adenosine-induced myocardial hyperemia is partly dependent on an intact endogenous NO production suggesting that adenosine-mediated vasodilation is partly endothelium dependent [20]. Thus, as we and others confirmed [21–25], a decrease in myocardial perfusion reserve may be caused by endothelial dysfunction.

There are different potential applications of this technique since CFR allows for the assessment of the hemodynamic relevance of a moderate and severe coronary stenosis, the detection of coronary restenosis, or upstream coronary occlusion. Moreover, evaluation of the CFR is of crucial importance in order to appreciate myocardial reperfusion or “no-reflow” following reopening of the infarct-related artery [2]. This technique gives also the possibility of noninvasive follow-up of arterial bypasses [2].

CFR measurement by transthoracic Doppler echocardiography reflects coronary microvascular function, as a cost-effective and noninvasive screening test in many conditions (age and sex, hypertension, diabetes, hypercholesterolemia, cardiomyopathies, valvular heart diseases, heart transplantation, endocrine, and immunitary diseases). Thus CFR represents a simple but at the same time very important tool to investigate the physiology and pathophysiology of heart and systemic diseases. Furthermore, it is also helpful in evaluating therapeutic interventions and prognosis-risk stratification in cardiomyopathies [26], coronary artery disease, and heart transplantation [2, 27–29].

Coronary microvascular dysfunction, defined as reduced coronary flow reserve and/or coronary endothelial dysfunction, is associated with a 2.5% annual major adverse event rate that includes death, nonfatal myocardial infarction, nonfatal stroke, and congestive heart failure [30]. Early identification of microvascular coronary disease by echo-CFR or other coronary reactivity tests may be beneficial in prognosis evaluation and patient stratification for optimal medical therapy [31]. This is of paramount importance because many diseases, that is, endocrine, metabolic, and immune conditions, affect vascular and in particular coronary function.

## 2. Hormonal Influences on Vascular Reactivity: At the Heart of Coronary Microvascular Dysfunction

**Figure 2:**
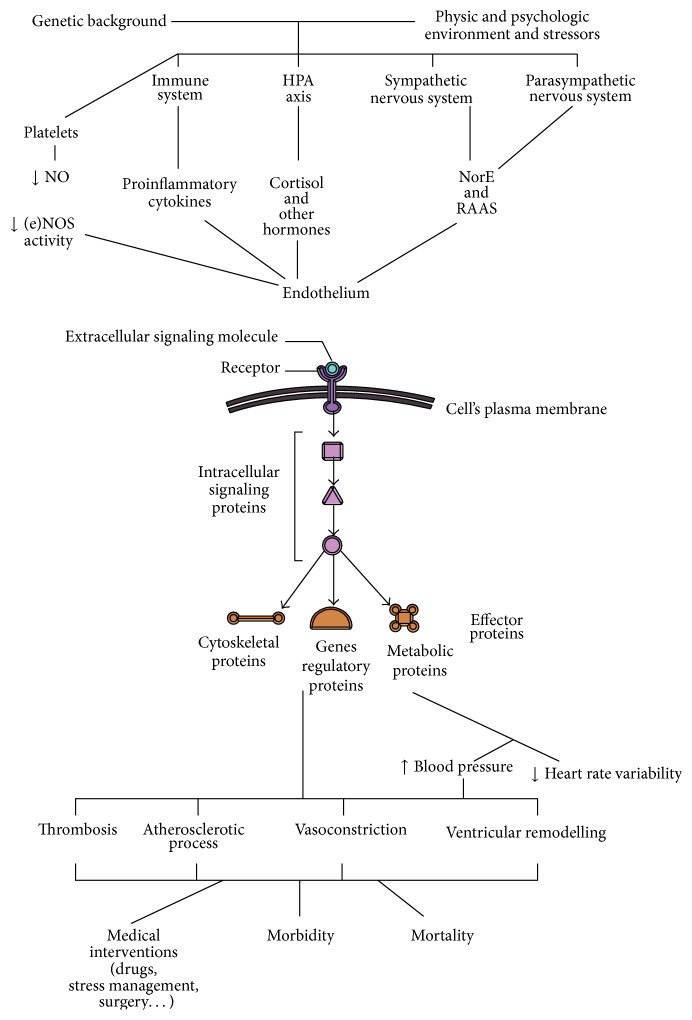
**“**Internal” and “external” stimuli activate and balance endocrine, immune, and psychoneurological responses. Cells, in the figure of endothelial cells, interact with the surrounding environment by interpreting extracellular signals via receptor proteins that span their membrane. Receptors are composed of extracellular and intracellular domain. The inner domain interacts with other intracellular signaling proteins that relay the message to one or more effector proteins. These proteins activate gene transcription and mediate different responses. (HPA axis: hypothalamus-pituitary-adrenal axis; NO: nitric oxide; (e)NOS: nitric oxide synthesis; NE norepinephrine; RAAS: renin-angiotensin-aldosterone system).

Endothelial cells integrate several different stimuli (Figure 2) to maintain the appropriate coronary tone and flow able to always match the variable myocardial demands. Here we describe the role of hormones and growth factors in this complex crosstalk.

### 2.1. Vitamin D

Vitamin D deficiency has been associated with prevalence and incidence of cardiovascular (CV) disease, suggesting a role for bioregulators of bone and mineral metabolism in CV health. In the absence of major cardiovascular risk factors, Vitamin D deficiency is a frequent finding in essential hypertension patients and is independently associated with left ventricular hypertrophy [32]. In this setting, endothelial Vitamin D receptor plays an important role in endothelial cell function and blood pressure control, regulating angiotensin II effects with its action being related to endothelial NO synthase expression [33].

Vitamin D deficiency leads to secondary hyperparathyroidism, a condition associated with CV disease [34]. Parathyroid hormone (PTH) is an important regulator of calcium homeostasis, and its elevation increases prevalence and incidence of CV risk factors and disease, including CV mortality and vascular structural abnormalities [34]. The higher risk persists after adjusting for Vitamin D levels, renal function, and standard risk factors. Thus, PTH represents an important CV risk factor with an independent predictive value for CV disease and mortality [35]. Furthermore, accumulating evidence suggests bidirectional interplay between PTH and aldosterone (which is a well-known independent CV risk factor [36]). This interaction may lead to a disproportionally increased risk of CV damage and metabolic and bone diseases. PTH stimulates aldosterone secretion by increasing the calcium concentration in the cells of the adrenal zona glomerulosa after binding to the PTH/PTH-rP receptor and indirectly by potentiating angiotensin-2 induced effects. This may explain why parathyroidectomy decreases aldosterone levels in parallel with improved cardiovascular outcomes. Aldosterone mediated effects are inappropriately pronounced in conditions such as chronic heart failure, excess dietary salt intake (relative aldosterone excess), and primary aldosteronism [37]. PTH is increased as a result of (1) the mineralocorticoid receptor (MR) mediated calciuretic and magnesiuretic effects and (2) direct effects of aldosterone on parathyroid cells via binding to the MR. Hyperparathyroidism causes myocardial fibrosis and disturbed bone metabolism. Furthermore, a link between Vitamin D, renin-angiotensin-aldosterone system, and the fibroblast growth factor 23/klotho pathways has been discovered recently and highlighted as an active cardiovascular regulator system [38, 39]. Hyperaldosteronism due to klotho deficiency results in vascular calcification, which can be mitigated by treatment with the aldosterone receptor antagonist spironolactone [40].

In asymptomatic primary hyperparathyroidism (PHPT), CFR was assessed as a marker of coronary microvascular function and inversely related to PTH levels. PTH independently correlated with the coronary microvascular impairment, suggesting a crucial role for the hormone to explain the increased cardiovascular risk in PHPT. Furthermore, coronary microvascular dysfunction in PHPT patients was completely restored after parathyroidectomy [21].

### 2.2. Thyroid Hormones

There is an association between subclinical hypothyroidism (SCH) and increased risk for cardiovascular disease as shown in a study performed by Traub-Weidinger et al. examining coronary vascular reactivity in asymptomatic patients with SCH before and after levothyroxine (LT4) supplementation [41]. In asymptomatic subjects with SCH due to thyroid autoimmunity, coronary microvascular function was impaired and improved after supplementation with LT4. These findings may explain the increased cardiovascular risk attributed to SCH [41].

Savinova et al. demonstrated that low thyroid hormone (TH) function influences coronary remodeling and reduces the density of small arterioles in heart failure. In fact, hypothyroidism results in arteriolar atrophy in the left ventricle. In this context, tri-iodothyronine (T3) treatment rapidly induces small arteriolar muscularization and, within 72 hours, restores arteriolar density to normal levels. T3 treatment results in the coordinate regulation of angiopoietin 1 and 2 expression. The response to angiopoietins leads to vessel enlargement. In addition to the effects of THs on vasoreactivity, these results suggest that THs may affect the function of small resistance arteries also influencing the remodeling of vascular smooth muscle cells [42].

Finally, TH is critical for living organisms when coping with environmental stress. Plasma circulating T3 levels drop in most disease states and are associated with increased oxidative stress [43, 44]. In this context, plasmatic T3 levels are an independent determinant for the recovery of cardiac function in patients after myocardial infarction [45]. Thyroid hormone receptor *α*1 (TR*α*1) seems to be crucial in this response. TR*α*1 translocates into the cell nucleus upon activation of stress induced growth kinase signaling. Furthermore, overexpression of nuclear TR*α*1 in cardiomyocytes can result in pathological or physiological growth (dual action) in absence or presence of its ligand, respectively. Accordingly, inactivation of TR*α*1 receptor preventing the reactive hypertrophy following myocardial infarction results in heart failure with increased phospholamban (PLB) expression and marked activation of p38MAPK [45]. In line with this evidence, TH limits ischemia/reperfusion injury and converts pathologic to physiologic growth after myocardial infarction via TR*α*1 receptor. In this view, TR*α*1 receptor may become a novel pharmacological target for cardiac repair/regeneration therapies [45].

### 2.3. Growth Hormone and Insulin Like Growth Factor-I

Growth hormone (GH) and insulin like growth factor-I (IGF-I) affect cardiac structure and performance. In the general population, low IGF-I has been associated with higher prevalence of ischaemic heart disease and mortality. Recent epidemiological evidence suggests that serum IGF-I levels in the low-normal range are associated with increased risk of acute myocardial infarction, ischaemic heart disease, coronary and carotid artery atherosclerosis, and stroke [46]. This confirms previous findings in patients with acromegaly or with GH-deficiency showing cardiovascular impairment [47]. Patients with either childhood- or adulthood-onset GHD have cardiovascular abnormalities such as reduced cardiac mass, impaired diastolic filling and left ventricular response at peak exercise, increased intima-media thickness, and endothelial dysfunction. These abnormalities can be reversed, at least partially, after GH replacement therapy [48].

CFR has been shown to decrease in adults with GHD. Direct correlation between CFR and IGF-1 concentrations suggests that GH replacement may improve microvascular function and likely decrease cardiovascular morbidity and mortality in patients affected by GHD [49].

On the contrary, in acromegaly, chronic GH and IGF-I excess causes a specific cardiomyopathy, that is, concentric cardiac hypertrophy (diagnosed in more than two-thirds of the patients) associated with diastolic dysfunction [47]. In later stages, impaired systolic function and heart failure can occur if GH/IGF-I excess is not controlled. Abnormalities of cardiac rhythm and of heart valves are also reported. Successful medical or surgical control of acromegaly is accompanied by the decrease of the left ventricular mass and improvement of cardiac function [48].

### 2.4. Cortisol

Coronary microvascular function, as assessed by CFR, is pathologically reduced in a considerable number of patients with Cushing's syndrome without clinical symptoms of ischemic heart disease and in the absence of epicardial coronary artery lesions [50]. Although the presence of comorbidities has to be taken into account to explain this early coronary abnormality in Cushing patients, CFR inversely relates to urinary cortisol in patients with endogenous hypercortisolism. Nevertheless, the possibility of using urinary cortisol as a predictor of coronary microvascular function in patients with Cushing's syndrome needs further investigation [50].

### 2.5. Sexual Hormones

Sexual hormones affect endothelial function. The protective effects of estrogens on cardiovascular function are well known. Females in their premenopausal period of life present a lower incidence of cardiovascular events in comparison with men of the same age and risk factor profile [51]. In postmenopause this difference tends to disappear. Estrogen administration in postmenopausal women is associated with a 50% reduction in the clinical manifestations of coronary artery disease [52]. One explanation seems to be the estrogen-induced modulation of coronary vasoreactivity. In fact, estrogens decrease basal coronary vasomotor tone as manifested by increased coronary flow, decreased resistance, and increased epicardial cross-sectional area. These hormonal actions on coronary vasoreactivity may explain, in part, the cardioprotective effects of estrogen in postmenopausal women [51, 53].

Epidemiological studies have shown a high prevalence of low serum testosterone levels in men with cardiovascular disease. Furthermore, low testosterone levels are associated in some, but not in all observational studies, with an increase in cardiovascular events and mortality [54]. Testosterone has beneficial effects on several cardiovascular risk factors, which includes cholesterol, endothelial dysfunction, and inflammation. Testosterone has vasodilatory actions on several vascular beds, although some studies have reported conflicting effects [54]. In clinical studies, acute and chronic testosterone administration increases coronary artery diameter and flow, improves cardiac ischemia and symptoms in men with chronic stable angina, and reduces peripheral vascular resistance in chronic heart failure. Testosterone is an L-calcium channel blocker and induces potassium channel activation in vascular smooth muscle cells. Animal studies [54] have consistently demonstrated that testosterone is atheroprotective, whereas testosterone deficiency promotes the early stages of atherogenesis; however, the mechanisms involved are not completely understood.

The role of testosterone on vascular function has been investigated also in heart transplanted patients. Among the various complications of heart transplantation (HTx), the vasculopathy of the allograft (CAV) represents a serious problem linked to chronic rejection [55]. Testosterone plasma levels may be involved in CAV development indirectly, increasing traditional risk factors and directly influencing the alloimmune response [55]. These findings have been confirmed assessing vascular erectile dysfunction (ED) and CFR in a group of heart transplanted patients. CFR was significantly reduced in ED versus no-ED patients and lower testosterone plasma levels were statistically associated with CAV [56]. These data about the effects of testosterone on endothelial function are in line with another study performed in a group of women with polycystic ovary syndrome (PCOS). Total testosterone, free testosterone, and androstenedione were increased in case of PCOS and CFR was preserved in this clinical condition [57].

Two recent studies raised some concerns about cardiovascular risks associated with testosterone therapy [58, 59]. Morgentaler et al. concluded that so far the available evidence of an increased cardiovascular risks induced by testosterone therapy is rather inconclusive and needs further appraisal [60].

### 2.6. Insulin and Glucagon-Like Peptide-1

Insulin has an important role in vascular function and recent evidence documents insulin-mediated vasodilatory effects at the level of coronary vessels [61]. In healthy subjects insulin enhances myocardial blood flow and decreases coronary vascular resistance in a dose-dependent manner [62]. Moreover, insulin is able to increase myocardial blood flow also in conditions characterized by coronary dysfunction such as obesity, diabetes type 1, and coronary artery disease. In contrast, hyperinsulinemia and insulin resistance, which are associated with an impaired vasodilatory effect of insulin, have been demonstrated to be an independent risk factor for coronary artery disease [63]. Hyperglycemia after oral glucose loading suppresses CFR in healthy young male subjects [64]. This result suggests that acute hyperglycemia may have adverse effects on coronary microcirculation. Instead, control or improvement of hyperglycemia improves CFR, as shown in poorly controlled diabetic patients [65].

Glucagon-like peptide-1 (GLP-1) has protective effects in the heart [66], mitigating coronary microvascular dysfunction through a reduction in oxidative stress [67]. The protective effects of GLP-1 are dependent on downstream inhibition of Rho through a cAMP/PKA-mediated pathway [68]. Moreover, GLP-1 regulates glucose-dependent insulin secretion and has numerous extrapancreatic effects, including the recruitment of cardiac muscle microvasculature in healthy humans [69].

### 2.7. Obesity

Obese patients have reduced myocardial vasoreactivity, which may represent an early precursor of future coronary artery disease. Insulin-induced enhancement of myocardial blood flow is blunted in obesity [70] but also serum leptin, a hormone produced by adipose tissue, is inversely related to the adenosine-stimulated myocardial flow suggesting a vasoactive role [71]. The early coronary microvascular impairment of obesity seems also to be related to a systemic chronic inflammation mediated by adipocytokines, independently of body max index [72]. Body weight loss improves coronary circulatory dysfunction [73] and bariatric surgery rapidly reverses obesity-induced endothelial dysfunction [74, 75] via a GLP-1-mediated mechanism [76].

### 2.8. Adipose Tissue and Adipokines

Fat cells surround coronary arteries and play a central although underrecognized role in the development of cardiovascular disease through the systemic secretion of adipokines. Adipokines are protein produced by the adipose tissue (especially by periadventitial adipose tissue) with paracrine and endocrine actions [77] able to regulate inflammatory molecule expression [78]. Spiroglou et al. showed that vascular dysfunction and atherosclerotic lesions are positively correlated with chemerin, vaspin, visfatin, and leptin fat expression at periaortic level [79]. Furthermore, coronary atherosclerosis positively correlates with chemerin and visfatin pericoronary fat expression and coronary flow impairment with chemerin and vaspin expression [80].

### 2.9. Vasopressin and Oxytocin

Vasopressin (VP) and oxytocin (OT) are mainly synthesized in the magnocellular neurons of the paraventricular and supraoptic nucleus of the hypothalamus and released into the blood from their axon projections in neurohypophysis. VP and OT act in complementary manner in cardiovascular control, as both hormones and neurotransmitters, regulating Ca^2+^ signaling [81]. While VP conserves water and increases circulating blood volume, OT eliminates sodium [82]. In most vascular beds VP is a potent vasoconstrictor [83], more potent than OT. The vasoconstriction by VP and OT is mediated via V1a receptor [84, 85]. Instead, in some vascular beds, such as the lungs and the brain, VP and OT produce NO dependent vasodilatation [86]. Martínez et al. studied the coronary effects of VP and its interaction with NO and prostanoids in a goat model, during partial ischemia and reperfusion. Ischemia led to the reduction of coronary vasodilatory reserve and attenuation of VP-induced vasoconstriction; the modulatory role of NO was preserved and there was a probable involvement of vasoconstrictor prostanoids. During reperfusion, the coronary vasodilatory reserve and the coronary reactivity to acetylcholine and VP are recovered [87].

Peripherally, VP has been found to enhance the sensitivity of the baroreceptor; instead centrally, VP and OT increase sympathetic outflow, suppress baroreceptor reflex, and enhance respiration [85]. While VP is an important mediator of stress followed by adrenocorticotropic hormone (ACTH) release, OT exhibits antistress properties [88]. Moreover, VP has been found to contribute considerably to the progression of hypertension and heart failure while cardiovascular actions of OT include lowering blood pressure, negative inotropic and chronotropic effects, parasympathetic neuromodulation, vasodilatation, anti-inflammatory activity, antioxidant activity, and metabolic effects [88]. OT actions are mediated by NO and ANP [89]. Recent evidence suggests that the enhanced stimulation of central angiotensin-1 and V1 receptors as well as the attenuated stimulation of oxytocin receptors accounts for the exaggerated cardiovascular responses to stress stimuli during the postinfarct state and that, on the contrary, angiotensin II, vasopressin, interleukin-1, and tumor necrosis factor-*α* (TNF-*α*) systems are important in the central cardiovascular control under resting conditions [90, 91]. In experimentally induced myocardial infarction, continuous* in vivo* OT delivery improves cardiac healing and cardiac work, reduces inflammation, and stimulates angiogenesis [86, 88, 92].

### 2.10. Prolactin

Recent studies show that hyperprolactinemia is associated with endothelial dysfunction, increased carotid intimal medial thickness, insulin resistance, and low-grade inflammation [93]. On the other hand, prolactin has been suggested to play an autocrine regulatory role in angiogenesis induced by the FGF2/STAT5 signaling cascade and VEGF expression induction [94].

Moreover, the treatment of porcine aortic endothelial cells by prolactin caused a reduction of NO production causing coronary, mesenteric, renal, and iliac vasoconstriction [95]. The stress hormone prolactin could be a costimulator of platelet activation in patients with acute coronary syndrome [96]. A pilot study showed that patients with prolactinoma are characterized by microvascular dysfunction as well as plasma markers indicating a proatherothrombotic state [97]. Prolactin is closely associated with autoimmune diseases in animal models and humans, and several disease-related autoantibodies were increased in patients with hyperprolactinemia [98]. Interestingly, the presence of antiendothelial cell antibodies may explain the prolactin's proinflammatory and proatherothrombotic effects [98].

The role of prolactin in vascular and myocardial regulation may be different according to the site of action and needs therefore to be better clarified.

### 2.11. Melatonin

Melatonin, the principal hormone of the vertebral pineal gland, exerts endothelial-dependent vasorelaxant effects, which potentiate significantly the effect of acetylcholine, and counteracts the vasoconstrictor responses to catecholamines [99]. These effects may be, in part, due to a melatonin favourable influence on the redox balance, with elevated NO and cGMP levels along with lower calcium in vascular tissue [100]. In animal models, melatonin decreases also the inflammatory factors acting on endothelial cells [101] and preserves capillary perfusion during ischemia-reperfusion events [102].

### 2.12. The Liver: Bilirubin, Heme Catabolic Pathway, and *γ*-Glutamyltransferase

Recent data has convincingly demonstrated that mildly elevated serum bilirubin levels are strongly associated with a lower prevalence of oxidative stress-mediated diseases [103]. Indeed, serum bilirubin has been shown to negatively correlate to CV diseases, as well as to CV risk factors such as arterial hypertension, diabetes mellitus, metabolic syndrome, and obesity. This data suggests a protective effect of bilirubin and of other products of the heme catabolic pathway such as biliverdin and carbon monoxide, as well as the key enzymes, heme oxygenase, and biliverdin reductase [103]. The heme-heme oxygenase system has recently been recognized to possess important regulatory properties: it is tightly involved in both physiological and pathophysiological processes, such as cytoprotection, apoptosis, and inflammation. Effects of the free heme on the vascular system are determined by extracellular factors, such as hemoglobin/heme-binding proteins, haptoglobin, albumin, hemopexin, and intracellular factors, including heme oxygenases and ferritin [104].

Serum *γ*-glutamyltransferase (GGT) levels are an independent risk factor for CV disease, and there is a strong association between serum GGT levels and most CV risk factors. In fact, high serum GGT levels, which correlate with low CFR, represent an independent marker of coronary microvascular damage and inflammation in normal individuals without concomitant risk factors [105].

## 3. Beyond CFR

### 3.1. Inflammation and Immune Function

Atherosclerosis has been identified as an inflammatory process [106] driven by the adaptive (T and B cells) immune system and dendritic cells [107]. Cardiac patients with increased levels of proinflammatory cytokines, such as TNF-*α* and IL-6, have increased risk of adverse clinical events [36]. Increased levels of proinflammatory cytokines are also found in heart failure patients likely as the consequence of cardiac remodeling [36]. We also documented a CFR reduction in young patients with severe psoriasis without coronary disease, suggesting that coronary microvascular dysfunction was an early complication of psoriasis independently related to the severity and extension of the skin manifestations and likely the consequence of the chronic systemic inflammation [108].

### 3.2. Platelet Function

Platelet abnormalities, such as increased activation or disturbed serotonin metabolism, causing platelet hyperreactivity and thrombosis, are linked to higher cardiovascular morbidity and mortality [36]. Even though myeloproliferative neoplasms are most commonly associated with venous thrombosis, up to 60% of patients experience a thrombotic event in their lifetimes, including stroke or myocardial infarction. We documented CFR reduction in asymptomatic patients with essential thrombocythemia and polycythemia vera [109]. These patients have coronary microvascular dysfunction in absence of clinical conditions suggesting CAD. Sickle cell disease is characterized by obstruction of microvessels leading to ischemia and necrosis and in this clinical setting an abnormal cardiac perfusion reserve is present [110]. Vasoocclusion represents a phenomenon involving endothelial cell dysfunction, leukocyte activation, platelet activation, and chronic inflammation resulting in multiple adhesive interactions between these cellular elements [111]. Since platelets mediate inflammation as well as thrombosis via release of pro- and anti-inflammatory molecules, they are crucial players to maintain cardiovascular health and to balance different neuroendocrine-immune signals [109].

### 3.3. Autonomic Balance

Stress, anxiety, and depression contribute to cardiovascular diseases including heart failure, ischemic disease, hypertension, and arrhythmias [112]. The appropriate balance (autonomic tone) between the sympathetic and the parasympathetic system, which are the two major components of the autonomic nervous system, is fundamental in the pathophysiology of cardiovascular diseases [112]. Chronic activation of the sympathetic system (which occurs in cases of chronic stress, depression, or anxiety, including also personality disorders) and/or decreased parasympathetic (vagal) tone is a hallmark of cardiovascular disease. The sympathetic system contributes to endothelial dysfunction, hypertension, and atherosclerosis [113, 114]. It promotes insulin resistance and dyslipidemia [115, 116] but also induces left ventricular hypertrophy [117], increases the incidence of arrhythmia, and promotes renal dysfunction by stimulating sodium and fluid retention [118], glomerulosclerosis, and the activation of the renin-angiotensin-aldosterone system (RAAS) [112, 119]. Chronic stress increases vascular responses to noradrenaline. This effect is endothelium-dependent and involves the release of vasoconstrictor prostanoids via stimulation of endothelial alpha-2 adrenoceptors [120]. Several lines of evidence support the role of inflammation and immune mechanisms in the pathogenesis of heart diseases [121] and endothelial dysfunction [122]. Interestingly, the nervous system can directly activate the immune system [123] and an inflammatory process may arise after the release of neuropeptides from nerves, in a process called “neurogenic inflammation” [124].

## 4. The Stress Response

Hans Selye's [125] inspired a huge and still growing wave of medical research. His experiments with rats led to the recognition of the “general adaptation syndrome,” later renamed by Selye “stress response”: the triad of enlarged adrenal glands, lymph node and thymic atrophy, and gastric erosions/ulcers [126].

**Figure 3:**
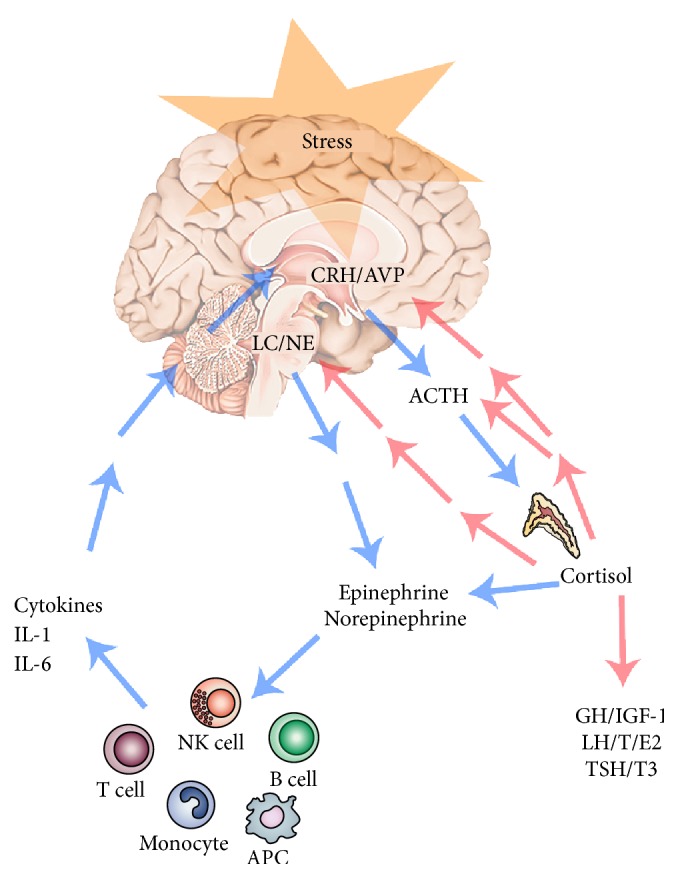
A simplified schematic representation of the stress system and the effectors of the stress response. The CRH/AVP neurons and central catecholaminergic neurons of the LC/NE system are reciprocally connected. Several feedback loops control the time-integrated secretion of cortisol and the activity of the HPA axis. Furthermore glucocorticoids stimulate the fear centers in the amygdala. Activation of the HPA axis leads to the suppression of the GH/IGF-1, LH/testosterone (T)/estradiol 2 (E2), and TSH/T3 axes. HPA axis, sympathetic system, and immune system activity are related. Blue lines indicate stimulation; red lines indicate inhibition. Abbreviations are as in the text. Modified from [130].

All organisms maintain a complex dynamic equilibrium, called homeostasis, which is constantly challenged by internal or external adverse forces termed “stressors” (hotness, coldness, toxins, infections, wounds, fatigue, psychosocial factors, etc.) [127]. Stress occurs when homeostasis is threatened or perceived to be so; homeostasis is reestablished by various physiological and behavioral adaptive responses that constitute the so-called “stress response” [128]. Thus stress could be defined, according to the original Selye's definition, as the general and nonspecific response to any request from the environment. Under favorable conditions, individuals can develop vegetative and pleasurable responses that enhance their emotional and intellectual growth and help the survival of their species, such as food intake and sex [129]. In contrast, activation of the stress response during threatening situations beyond the control of the individual can be associated with dysphoria and eventually emotional or somatic disease [130]. Tsigos and Chrousos reviewed the mechanisms underlying the stress response [130] (Figure 3). Briefly, “the main components of the stress system are the corticotropin-releasing hormone (CRH) and locus ceruleus-norepinephrine- (LC/NE-) autonomic systems and their peripheral effectors, the pituitary-adrenal axis, and the limbs of the autonomic system. An active stress system leads to behavioral and peripheral changes that improve the ability of the organism to adjust homeostasis and increase its chances for survival. The CRH and LC/NE systems stimulate arousal and attention, as well as the mesocorticolimbic dopaminergic system, which is involved in anticipatory and reward phenomena, and the hypothalamic beta-endorphin system, which suppresses pain sensation and, hence, increases analgesia. CRH inhibits appetite and activates thermogenesis via the catecholaminergic system. Moreover, reciprocal interactions exist between the amygdala and the hippocampus and the stress system, which stimulates these elements and is regulated by them. During stress CRH inhibits GnRH and, through somatostatin, GH, TRH, and TSH secretion, which in turn, suppress the reproductive, growth, and thyroid functions. Interestingly, all these functions receive and depend on positive catecholaminergic input. The hormones at the end of the hypothalamic-pituitary-adrenal (HPA) axis and glucocorticoids have multiple roles. They simultaneously inhibit the CRH, LC/NE, and b-endorphin systems and stimulate the mesocorticolimbic dopaminergic system and the CRH peptidergic central nucleus of the amygdala. In addition, they directly inhibit pituitary gonadotropin, GH, and TSH secretion, render the target tissues of sex steroids and growth factors resistant to these substances, and suppress the 50 deiodinase, which converts the relatively inactive tetraiodothyronine (T4) to triiodothyronine (T3), contributing further to the suppression of reproductive, growth, and thyroid functions. They also have direct as well as insulin-mediated effects on adipose tissue, ultimately promoting visceral adiposity, insulin resistance, dyslipidemia and hypertension (metabolic syndrome X), and direct effects on the bone, causing ‘‘low turnover” osteoporosis. Central CRH, via glucocorticoids and catecholamines, inhibits the inflammatory reaction, while directly secreted by peripheral nerves CRH stimulates local inflammation [124] (immune CRH)” [130].

As demonstrated by Charmandari et al., appropriate responsiveness of the stress system to stressors is a crucial prerequisite for a sense of wellbeing, adequate performance of tasks, and positive social interactions. By contrast, inappropriate responsiveness of the stress system may impair growth and development and may account for a number of endocrine, metabolic, autoimmune, and psychiatric disorders [131, 132]. The development and severity of these conditions primarily depend on the genetic vulnerability of the individual, the exposure to adverse environmental factors, and the timing of the stressful events, given that prenatal life, infancy, childhood, and adolescence are critical periods characterized by increased vulnerability to stressors [131, 133]. A hyper- or hypoactive stress system associated with abnormalities of the systemic anti-inflammatory feedback and/or hyperactivity of the local proinflammatory factors play a relevant role in the pathogenesis of chronic inflammation and immune-related diseases, such as atherosclerosis, hypertension ischaemic heart diseases, or heart failure [132].

### 4.1. Mental Stress Ischaemia

There is a great amount of literature on psychological stress (see “stress response” below) and cardiovascular disease [134]. Studies about the effects of acute stressors have been performed in people that experienced disasters (earthquakes or hurricanes) [135, 136] while studies about chronic stressors evaluated, for example, the effects of job stress [137], marital unhappiness [138, 139], and burden of caregiving [140]. From all of these studies there are extensive data concerning stressors' contributions to diverse pathophysiological changes including sudden death, myocardial infarction, myocardial ischemia, and wall motion abnormalities, as well as to alterations in cardiac regulation as indexed by changes in sympathetic nervous system activity and hemostasis [140]. The concept of “personality” is intimately linked to the concept of mental stress. Some personality patterns [141], that is, depressive or aggressive moods, are linked with higher incidence of cardiovascular diseases through chronic stress axis activation [36, 142]. Mostofsky et al. described outbursts of anger as a trigger of acute cardiovascular events [143]. Prolonged impairment of endothelial function has been documented in healthy men after a brief episode of mental stress [144, 145] and CFR reduction [146, 147]. The Takotsubo cardiomyopathy (or “stress cardiomyopathy”) consists of a transient left ventricular dysfunction triggered by acute emotional or physical stress, whose clinical presentation mimics acute myocardial infarction, with acute chest pain, transient ST elevation, and apical ballooning on echocardiography. Although its cause remains elusive, coronary artery vasospasm, coronary microcirculation dysfunction [148], obstruction of the left ventricular outflow tract (LVOT), and catecholamine overload have been proposed as mechanisms for the injury. Myocytolysis and the histopathological lesions observed in takotsubo cardiomyopathy have been related with several brain conditions and neurogenic mechanisms of cardiac disease like catecholamine infusion, brain stimulation, and stress. Interestingly, evidence shows that heart lesions may occur even in adrenalectomized animals (although less pronounced), which suggests that these findings may be dependent on the direct action of nerve terminals in the heart [112]. All these considerations may explain the lack of efficacy of beta-blockade on low heart rate-related ischemia during mental stress [149] and highlight the importance of stress management in prevention and treatment of cardiovascular diseases [140].

### 4.2. Cellular Signaling Pathways

Generally, cells involved in regulating CV homeostasis respond to changes in their local environment using a range of receptors, among which the G-proteins coupled receptors are the most important. A signal recognition is then transformed into a cellular response (physiological or pathological) through intracellular transduction mechanisms that converge on the regulation of the phosphorylation state of intracellular proteins by a range of protein kinase and protein phosphatase enzymes (Figure 2). It seems likely that subtle defects in these mechanisms may lead to a number of cardiovascular pathologies. The high complexity of these signaling systems allows various cells to act in concert to maintain homeostasis responding rapidly to small and fluctuating changes in the incoming environmental signals, while the crosstalk between signaling pathways allows coordinated responses to multiple different and sometimes opposing signals [150, 151]. As an example, the receptor for the epidermal growth factor (EGF) and related ligands (EGFR), the prototypal member of the superfamily of receptors with intrinsic tyrosine kinase activity, is widely expressed on many cell types, including epithelial and mesenchymal lineages [152]. Upon stimulation by at least five genetically distinct ligands (including EGF, transforming growth factor-*α* (TGF-*α*), and heparin-binding EGF (HB-EGF)), the intrinsic kinase is activated and EGFR tyrosyl phosphorylates itself and numerous intermediary effector molecules, including closely related c-erbB receptor family members [153]. This step initiates multiple signaling pathways, some of which are involved in negative feedback loops [154]. The integrated biological responses to EGFR signaling are pleiotropic including mitogenesis or apoptosis, enhanced cell motility, protein secretion, and differentiation or dedifferentiation [155]. EGF network has a critical role in normal heart function and in normal cardiac valve formation in conjunction with ErbB receptors [156]. Moreover, current evidence suggests that angiotensin II receptor mediates transactivation of the EGF receptor on cardiac myocytes involving stimulation of the activities of a family of membrane-associated metalloprotease enzymes [150]. These enzymes ultimately cleave EGF receptor ligands (such as heparin-binding EGF) from their membrane-associated precursors; once released these ligands can stimulate EGF receptors leading to the activation of several signaling pathways in the myocytes [150]. Phosphatase and kinase activities regulate a number of cytosolic and nuclear phosphorylation events which together control the myocardial gene transcription involved in ventricular hypertrophy [150].

Understanding which mechanisms activated by extracellular stimuli are able to modify the cardiac and vascular cell functions will allow a deeper insight into the pathophysiology of cardiovascular disease. This knowledge will lead to the identification of novel molecular targets for pharmacological intervention and will assist the future development of therapeutic strategies against cardiovascular disorders.

## 5. The Heart and Cardiovascular System in the “Psychoneuroendocrine Immunologic” Network

Psychoneuroendocrine immunology studies the interactions among behavioral, neural and endocrine, and immunologic processes of adaptation [157]. The exploration of the extensive interactions among psychological and behavioral factors, the nervous system, the immune system, and the endocrine system may help understand the mechanisms underlying health, wellness, and diseases [158]. Many studies evidences have supported the close relationships between stress, depression, inflammation, and disorders including diabetes, obesity, and cardiovascular disease [159].

Psychological and nervous factors act on the cardiovascular system. The immune system is primarily involved in the pathological processes leading to left ventricular dysfunction and fibrosis [160] and communicates with the nervous and endocrine systems to maintain cardiac homeostasis [147].

The hormonal influence on the heart extends beyond endothelial function. For example, both cardiac myocytes and cardiac stem cells express the growth hormone releasing hormone receptor (GHRH), whose activation improves injury responses after myocardial infarction, reversing ventricular remodeling and enhancing heart functional recovery [161]. We already discussed the action of T3 on cardiomyocytes via TR*α*1. Furthermore, parathyroid hormone improves contractile performance of adult rat ventricular cardiomyocytes at low concentrations [162]. Growth factors that stimulate proliferation of fetal cardiomyocytes include angiotensin II, cortisol, and IGF-1. Two normally circulating hormones, atrial natriuretic peptide and T3, suppress cardiomyocytes proliferation [163].

Furthermore, the heart acts as a gland, for example, secreting atrial natriuretic peptide, and as an immune relay [164] which gives afferent neurological input to the brain [165, 166]. There is dynamic, bidirectional communication between the heart and brain influencing reciprocal functions. The heart communicates with the brain in four major ways: neurologically (through the transmission of nerve impulses) and electrically [167], biochemically (via hormones and neurotransmitters), and biophysically (through pressure waves).

## 6. Conclusion

**Figure 4:**
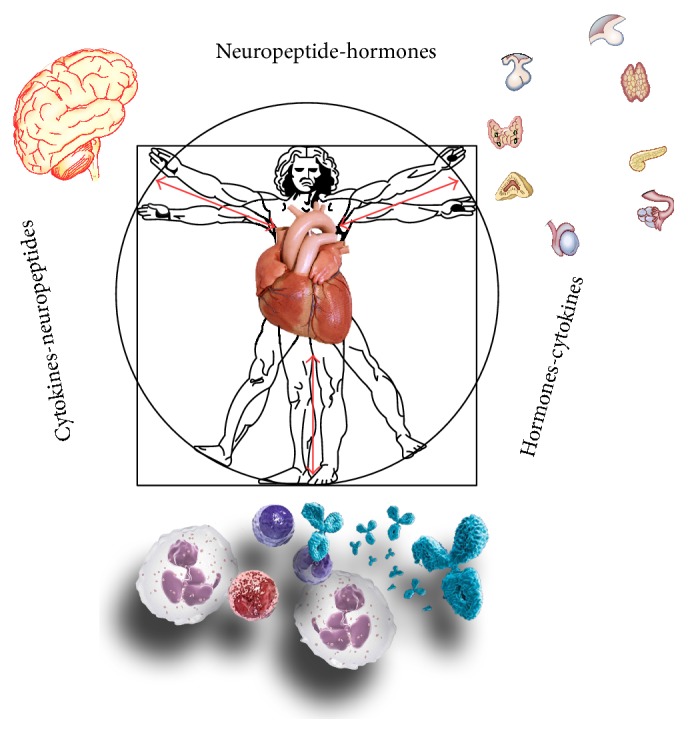
The cardiovascular system in the psychoneurological, hormonal, and immune network: it receives and sends signals to the brain, to the immune, and to the endocrine system [75]. As in Leonardo's Vitruvian man, also this network represents harmony and perfection in proportions. Psyche, brain and nerves, glands and hormones, lymphocytes, and cytokines are synchronized, they interact with each other, and the harmony between these systems involves and regulates cardiovascular functions.

In the present review, we depicted how psychoneurological, hormonal, and immune functions affect and regulate cardiovascular homeostasis and in particular coronary function (Figure 4). Nowadays, the cardiovascular system is conceived as the centre of a complex multiple organ network in which all components contribute to our health and wellbeing [168]. In this view, integrative medicine has emerged as a new therapeutic model that is patient centered and healing oriented [169]. Such patient care emphasizes the therapeutic relationship and uses therapeutic approaches originating from both conventional medicine and alternative medicine, such as meditation [170–172], music listening [173, 174], alimentation [175, 176], or physical exercises [177, 178]. All these lifestyle and behavioral aspects, counteracting the stress response, have a positive effect on our health and on our cardiovascular system [179]. In this context, the regulation of coronary reserve function clearly shows the importance of psychoneuroendocrine-immunitary factors in physiological but also pathological conditions and suggests the need to explore new therapeutic horizons against coronary artery disease.
